# Assessment of Disinfection Potential of Q-Switch Nd: YAG Laser on Contaminated Titanium Implant Surfaces

**DOI:** 10.3390/ma14206078

**Published:** 2021-10-14

**Authors:** Melanie Namour, Marwan El Mobadder, Baudouin Mulongo, Olivier Fagnart, Assaf Harb, André Peremans, Tim Verspecht, Wim Teughels, Samir Nammour, Eric Rompen

**Affiliations:** 1 Department of Dental Sciences, Faculty of Medicine, University of Liege, 4000 Liege, Belgium; melanienamour@gmail.com (M.N.); marwan.mobader@gmail.com (M.E.M.); eric.rompen@chuliege.be (E.R.); 2 Laboratoire de Microbiologie CEBIODI, Hospital Saint Jean, 32, Boulevard du Jardin Botanique, 1000 Bruxelles, Belgium; bmulongo@clstjean.be (B.M.); o.fagnart@clstjean.be (O.F.); 3 Laboratoire CEBIODI, Hospital Saint Anne, Saint Remi, 1070 Brussels, Belgium; Assaf.Harb@chirec.be; 4 Laboratoire Physique de la Matière et du Rayonnement, Université de Namur, 5000 Namur, Belgium; Andre.peremans@gmail.com; 5 Department of Oral Health Sciences, University of Leuven (KU Leuven), Kapucijnenvoer 33, 3000 Leuven, Belgium; tim2.verspecht@skynet.be; 6 Department of Oral Health Sciences, Dentistry, University of Leuven (KU Leuven), University Hospitals Leuven, Kapucijnenvoer 33, 3000 Leuven, Belgium; wim.teughels@kuleuven.be

**Keywords:** peri-implantitis, disinfection, titanium, implant, periodontology, dental implant, laser

## Abstract

Peri-implantitis (PI) is a relatively frequent pathology that compromises the overall survival of the dental implant. Adjunctive approaches for the conventional mechanical debridement are being suggested to optimize the treatment of PI. The goal of the study was the assessment of the disinfection potential of the Q-Switch Nd: YAG laser on contaminated titanium implant surfaces. A total of 72 sterile titanium discs were used and divided into three groups: 24 contaminated titanium discs treated with the laser (study Group L), 24 contaminated titanium discs with no treatment (control 1—Group C), and 24 sterile titanium discs with no treatment (control 2—Group S). Multi-species biofilm was used: Porphyromonas gingivalis, Fusobacterium nucleatum, Aggregatibacter actinomycetemcomitans, Streptococcus mutans, Streptococcus sobrinus, and Prevotella intermedia. Commensal bacteria were included also: Actinomyces naeslundii, Actinomyces viscosus, Streptococcus cristatus, Streptococcus gordonii, Streptococcus mitis, Streptococcus oralis, Streptococcus sanguinis, Streptococcus parasanguinis, and Veillonella parvula. Parameters delivered per pulse on the targeted surfaces of the titanium discs were an energy density of 0.597 J/cm^2^ each pulse, a pulse power of 270 mW, a laser beam spot of 2.4 mm in diameter, and a rate of repetition of 10 Hertz (Hz) for a pulse duration of 6 nanoseconds (ns). The mode was no contact, and a distance of 500 micrometers was used with a total time of irradiation equal to 2 s (s). The collection of microbiological samples was made for all groups; colony-forming units (CFU) were identified by two different practitioners, and the average of their examinations was considered for each sample. The average of the TBC (CFU/mL) was calculated for each group. Values were 0.000 CFU/mL, 4767 CFU/mL, and 0.000 CFU/mL for Group L, Group C, and Group S, respectively. Therefore, the suggested treatment protocol was able to provoke a total disinfection of the contaminated titanium surfaces. A statistical difference was only found between Group L vs. Group C and between Group S vs. Group C. The difference was not significant between Group S and Group L. In conclusion, the present study confirmed that the Q-Switch Nd: YAG laser under our specific conditions can provide a total disinfection of the contaminated titanium surfaces.

## 1. Introduction

The maintenance of a healthy peri-implant tissue presents one of the major challenges in dentistry for both practitioner and patient [1,2,3]. Peri-implant health can be categorized clinically by the absence of inflammatory tissues surrounding the dental implant. In this context, Araujo et al. [2] concluded in the 2017 world workshop on the classification of periodontal and peri-implant diseases and conditions, co-presented by the American Academy of Periodontology (AAP) and the European Federation of Periodontology (EFP) that a healthy peri-implant tissue presents a healthy mucosa comprised of a core of connective tissue covered by keratinized or non-keratinized epithelium and healthy intrabony tissue comprised of an implant in contact with mineralized bone [2]. Deviation from these features of the healthy peri-implant tissue may lead to two pathologies defined as peri-implant mucositis and peri-implantitis (PI) [4,5].

Various definitions of PI are present in literature. Kotsovilis et al. outlines peri-implantitis as the presence of peri-implant pocket depth greater than 5 mm around the implant, with bleeding on probing and a vertical bone loss [6]. The EFP in the 7th workshop defined peri-implantitis as a chronic inflammation of the peri-implant tissue with bleeding on probing and a marginal bone that has a vertical distance of more than 2 mm from the original marginal bone level obtained after the remodeling of the bone or a modification of the bone level with the presence of a periodontal pocket depth [7]. These numerous definitions of PI have led to a difficulty in the comparison of studies’ results.

It is agreed that PI is a complex pathology with similarities to periodontitis, having the bacterial infection as a main etiology and being largely dependent on the host response [8]. In addition, PI present a prevalence of 22% per implant and 19% per patient, which can be considered as relatively high [8,9]. Despite the similarities with periodontitis, it is demonstrated that PI presents a slight difference in bacterial component. In fact, PI sites present more often the aggregatibacter actinomycetemcomitans bacteria, Prevotella intermedia, Fusobacterium nucleatum, and Porphyromonas gingivalis [10,11].

Concerning the management of PI, Lang et al. [12] suggested a decisional tree known as the Cumulative Interceptive Supportive Therapy (C.I.S.T) that begins with non-invasive approaches consisting of mechanical debridement and oral hygiene instruction for pockets with a depth less than 3 mm. Meanwhile, more invasive treatments were suggested for pockets with depth greater than 5 mm, consisting of resective surgeries to re-establish a healthy periodontium. These invasive approaches include osteotomy, gingivectomy, and regenerative surgeries with or without guided tissue regeneration and/or bone grafting and other techniques [12]. However, to this day, there is no standardized treatment protocol for the management of PI [12].

In similarity with the management of periodontitis, assisted protocols are needed to optimize the outcome of the conventional mechanical debridement. Some of these approaches are showing promising results with a significant improvement in clinical periodontal parameters such as the use of antibiotics [13], chlorhexidine (CHX) [14], antimicrobial therapy [15], probiotics [16,17], antimicrobial peptides [18], and lasers [19,20].

Among the available approaches in the literature, Namour et al. [21] showed that the Q-switch Nd:YAG laser is able, when used with specific irradiation protocol and parameters (0.597 J/cm^2^; 10 Hz, pulse duration of 6 nanoseconds), to accomplish an effective photo-desorption of the contaminants of the titanium implant surfaces with a minimal heat generation surrounding targeted surfaces around 1 °C that was considered to be below the threshold of bone damage [21]. The authors have made a theoretical modeling and calculation of the optimal laser fluency emitted in nanoseconds that can increase the temperature of titanium surfaces to allow the desorption of calculus, organic materials, and debris [21]. This resulted in the total absence of carbon and without causing any morphological change of the irradiated implant surfaces. In a later study, Namour et al. [22] demonstrated that similar irradiation protocol with the Q-switch Nd: YAG laser was able to significantly eliminate the multi-species biofilm from the titanium surface [22]. The authors did not assess the disinfection state of that irradiated titanium surfaces. However, a total disinfection of the titanium surface is necessary for the osteointegration and the regeneration of the periodontium.

Therefore, the aim of this study is to assess the disinfection potential of a Q-Switch Nd: YAG laser on contaminated titanium surfaces. The null hypothesis is that no significant difference will be found in the level of disinfection between contaminated but untreated titanium surfaces and those treated with laser.

## 2. Materials and Methods

### 2.1. Study Design

**Figure 1:**
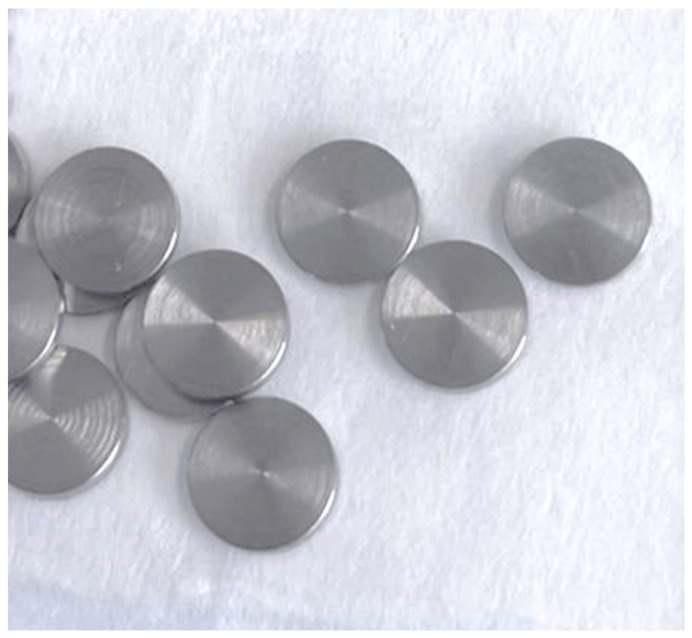
View of the titanium discs before the experiments.

**Figure 2:**
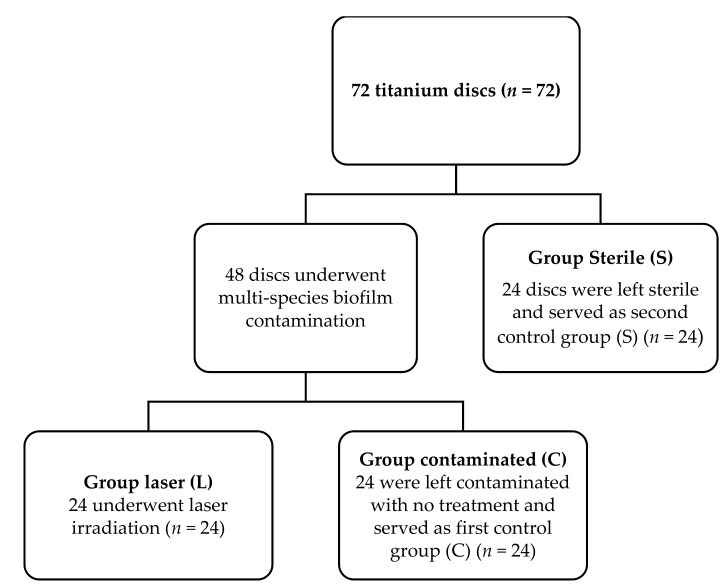
Design of the study. Group L is the study group consisting of contaminated titanium discs + treated with the laser; Group C is the control group consisting of contaminated discs with no treatment; Group S is the second control group consisting of sterile titanium discs with no treatment.

Seventy-two sterile titanium grade 4 discs having a surface composition of 15% zircon and 85% titanium (Roxolid^®^, SLActive, Straumann dental implant, Straumann AG, Basel, Switzerland) were used in this study. The size of each titanium disc was 5 mm in diameter and 1 mm in thickness (Figure 1). The titanium discs were divided into three groups: one study group and two control groups. An in vitro study does not require prior approval from the ethical committee of the University of Liège. The three groups of the study were divided as following (Figure 2).

#### 2.1.1. Group L

Twenty-four titanium discs were contaminated with multi-species biofilm and then irradiated with the Q-switch Nd:YAG laser according to the protocol of the study and were considered as the study group (Group L; Study group; *n* = 24).

#### 2.1.2. Group C

Twenty-four titanium discs were contaminated with multi-species biofilm and were left without any treatment and served as the first control group (Group C; *n* = 24).

#### 2.1.3. Group S

Twenty-four titanium discs were kept sterile, did not receive any kind of treatment, and were considered as the second control group (Group S; *n* = 24).

### 2.2. Contamination of the Titanium Discs with Multi-Species Biofilm

#### 2.2.1. Bacterial Straining, Media and Growing Settings

The fifteen multi-species biofilm that were used to contaminate the titanium discs are the essential bacterial strains found in peri-implantitis sites. The included bacterial strains were Porphyromonas gingivalis (ATCC 33277), Aggregatibacter actinomycetemcomitans (ATCC43718), Prevotella intermedia (ATCC 25611), Fusobacterium nucleatum (ATCC10953), Streptococcus mutans (ATCC 25175), and Streptococcus sobrinus (ATCC 33478). Commensal bacteria were also included in the study in order to mimic as possible the peri-implant biofilm. The commensal bacteria were Actinomyces viscosus (ATCC 15987), Streptococcus cristatus (ATCC 49999), Actinomyces naeslundii (ATCC 51655), Streptococcus mitis (ATCC 49456), Streptococcus oralis (DSM 20627), Streptococcus sanguinis (LMG 14657), Streptococcus gordonii (ATCC 49818), Veillonella parvula (DSM 2008), and Streptococcus parasanguinis (DSM 6778).

- All included multi-species were grown on an agar blood (Oxoid, Ltd., Basingstoke, UK) added by means of 1 µg/mL menadione, 5 µg/mL hemin (Sigma-Aldrich Co, St.-Louis, MO, USA), and 5% of horse blood that is sterile (E&O Laboratories Ltd., Bonnybridge, UK).
- Actinomycetemcomitans, S. cristatus, S. mitis, S. gordonii, S. oralis, S. mutans, S. parasanguinis, S. salivarius, S. sobrinus, and S. sanguinis were grown at 37 °C in a 5% carbon dioxide (CO_2_) environment. As for the Actinomyces naeslundii, Actinomyces viscosus, F. nucleatum, P. gingivalis, P. intermedia, and V. parvula, they were grown at 37 °C in anaerobic conditions (80% diazote, ten percent dihydrogen, and 10% CO_2_).
- Blood agar plates were used to prepare and collect the single species planktonic cultures. Afterwards, the prepared species were injected in 10 mm brain–heart infusion broth (BHI) (Difco Laboratories, Detroit, MI, USA) and then incubated under identical conditions as the blood agar plates, depending on bacterial species. Spectrophotometry (OD_600_, Gene Quant Spectrophotomoeter, Biochrom Ltd., Cambridge, UK) was used to assess the optical densities at 600 nm.
- Multi-species biofilms were grown in modified BHI broth, consisting of 37 g/L BHI added with 2.5 g/L mucin from porcine stomach type-III (Sigma-Aldrich Co, St.-Louis, MO, USA), 1.0 g/L yeast extract (Oxoid, Basingstoke, UK), 0.13 gram per liter cysteine HCl (Merck-Calbiochem, San Diego, CA, USA), 2.0 g/L sodium bicarbonate (Merck, Darmstadt, Germany), and 3.65 g/L 0.25% glutamic acid (Merck-Calbiochem, San Diego, CA, USA). Multi-species biofilms were grown at 37 °C with microaerophilic circumstances (6% O_2_, 7% CO_2_, 7% H_2_, and 80% N_2_).

#### 2.2.2. Bioreactor-Derived Multi-Species Biofilms and Community

The multi-species communities were established in a bioreactor (BIOSTAT^®^ B- Benchtop Bioreactor Controller, Sartorius BioTech GmbH, Gottingen, Germany) through a vessel containing seven hundred and fifty milliliters of modified BHI broth supplemented in addition to 5 milligrams per liter, 1 milligram per liter, and 200 microliters per liter of hemin, menadione, and Antifoam Y-30, respectively. The medium was nonstop stirred (300 rpm) at a fixed temperature of 37 degrees Celsius that is bubbled with 100% N2, 5% carbon dioxide, and a pH set around 6.7 with an interval of 0.1 plus or minus. After twenty-four hours, an ON culture of S. mitis was inoculated inside the vessel and grown until the late exponential stage. After that, the remaining bacterial ON cultures were carefully adjusted to an OD_600_ of almost 1.2 to 1.4 and further added to the vessel. A 14-species community was allowed to establish with a time of almost 48 h and was later kept in continuous culture by exchange of two-hundred milliliters of medium every twenty four hours [23]. In the study of Slomka et al., the exact protocol and the exact parameters that were used can be found [23].

Horizontally on the discs of titanium and on the bottom of a forty-eight well plate, the included multi-species biofilms that were already mentioned were grown. The included samples that were obtained from the bioreactor-derived community underwent 10-fold dilution in adapted brain infusion medium that was kept fresh; afterward, five hundred microliters were added to respectively each one of the titanium discs. During a time of forty-eight hours, the biofilm were permitted to establish in microaerophilic conditions (7% CO_2_, 6% O_2_, 7% H_2_, and 80% N_2_) at 37 °C and 170 rpm.

**Figure 3:**
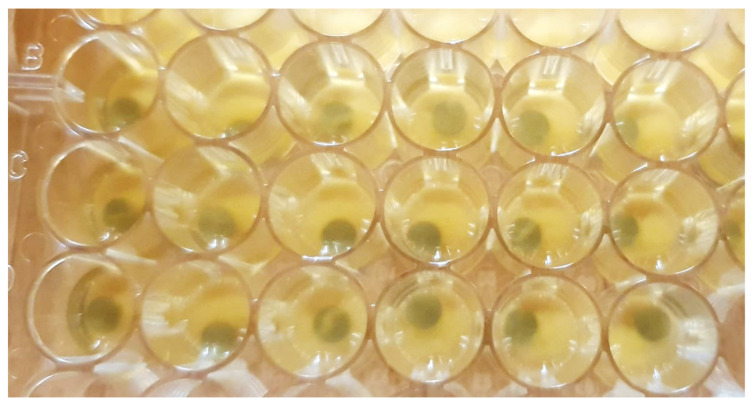
View of titanium discs with biofilm immersed in a suitable nutrient medium (Group C) before the experiments and the microbiological assessment.

Titanium discs containing the biofilms were dip-rinsed in phosphate-buffered saline with a 7.4 pH to remove all cells that can be found unattached. Subsequently, the titanium discs were moved to a new 48-well plate and then subjected to experimentations (Figure 3).

### 2.3. Laser Protocol and Irradiation Parameters 

The irradiation parameters used in this study were similar to the irradiation parameters used by Namour et al. [21,22] based on the theoretical modeling of the optimal energy density respecting the implant surfaces’ porosity.

In this study, a 1064 nanometers (nm) wavelength Q-switch Nd: YAG laser (Q-smart 850, Lumibird, Lannion, France) was used. Before irradiation and in order to evaluate the exact energy delivered and to calibrate it, a wattmeter (Ophir Spiricon Europe GmbH, Darmstadt, Germany) was used. The following irradiation parameters were delivered per pulse on the targeted surfaces of the grade IV titanium discs: an energy density of 0.597 Joules per cm^2^ per pulse, and a power per pulse of 270 mW. The spot size of the laser beam was 2.4 mm in diameter at irradiated surfaces. The repetition rate was ten Hertz (10 Hz) for a pulse duration of 6 nanoseconds (ns). During irradiation, the laser was placed in no-contact mode, and the distance was 500 micrometers. The total time of irradiation was equal to 2 s. At the end of irradiation, a microbiological sample was taken from each titanium disc.

### 2.4. Microbiological Assessment

**Figure 4:**
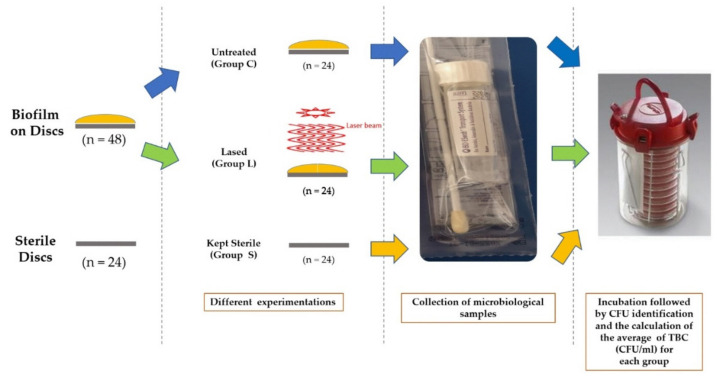
Schematic representation of the different steps and groups of the experiment.

The collection of microbiological samples was made for all groups: contaminated discs but not treated (Group C, control 1), contaminated discs + treated with laser (Group L), and sterile discs (Group S, control 2). The samples were taken carefully from each disc surface according to the instruction manual enclosed with a sterile Becton ESwabs™ (Becton, Dickinson and Company, Franklin Lakes, NJ, USA). An ESwa*b* combines a COPAN-invented flocked swab with one millimeter of Liquid Amies (non-nutritive balanced salt solution containing inorganic phosphates) in a plastic, screw-cap tube for a collection and transport system. From each sample, 10 µL of Liquid Amies were plated onto Schaedler agar (Becton, Dickinson and company, Franklin Lakes, NJ, USA) and incubated at 37 °C for 72 h in anaerobic medium of a gas mixture (H2: 10%, CO_2_: 10%, and N_2_: 80%). The identification of bacteria was performed by mass spectrometry (MALDI-TOF, Bruker Labscape, Bruker Belgium S.A./N.V, Kontich, Belgium). Colony-forming units (CFU) were identified by two different practitioners, and the average of each examination values was considered for each sample. The CFU grown were counted and transformed into actual total bacteria counts (TBC) based on 100-fold dilution factor. The average of the TBC (CFU/mL) was calculated for each group (Figure 4).

### 2.5. Statistical Analysis

The software that was used to do the statistical analysis was Sigma five^®^ (GraphPad Prism 5, San Diego, CA, USA). Statistical significance was considered when P was less than 0.05. The confidence level of the study was proposed to be 99% with a P value that is lower than 0.001; therefore, there was a very high significance. Mean and standard deviations (Std) were considered. Smirnov and Kolmogorov tests were utilized to assess the normality tests. One-way ANOVA coupled with a Newman–Keuls multiple comparison test (post hoc test) were used.

## 3. Results

**Figure 5:**
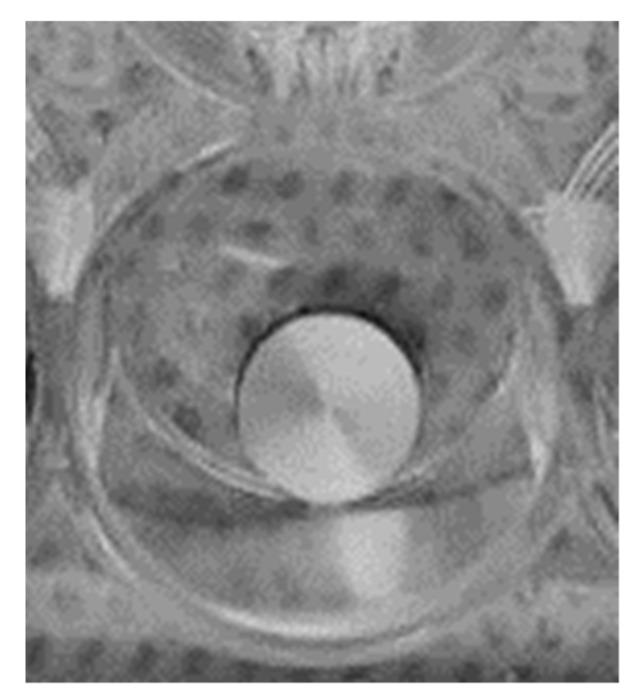
Visual appearance of a titanium disc immediately at the end of the irradiation protocol. This disc had been contaminated and treated by the Q-Switch Nd: YAG laser (Group L).

The mean values of TBC (CFU/mL) of the microbiological assessment were respectively 0.000 CFU/mL, 0.000 CFU/mL, and 4767 ± 1924 CFU/mL for the group of untreated sterile titanium discs (Group S), the group of contaminated titanium discs that have been irradiated (Group L), and the group of contaminated and untreated titanium discs (Group C). The visual appearance of contaminated titanium discs that were irradiated (Group L) showed no critical aspect change (Figure 5).

**Figure 6:**
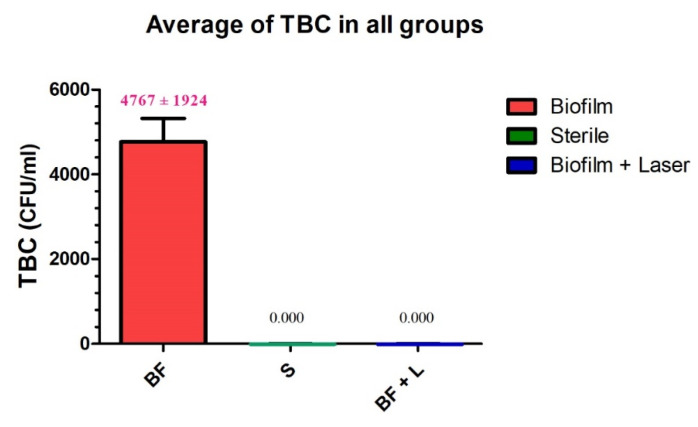
Results of the microbiological assessment in the three groups.

All values of all groups passed the normality tests. There was no statistically significant difference between the contaminated titanium surfaces that were treated with the Q-switch Nd: YAG laser (Group L) and the sterile titanium discs (Group S). On the other hand, there was a high statistically significant difference between the contaminated and untreated titanium discs (Group C) and the group of contaminated titanium discs that were irradiated (Group L). Likewise, there was a high statistically significant difference between the group of sterile titanium discs that were left untreated (Group S) and the contaminated and untreated titanium discs (Group C) (Table 1, Figure 6). Hence, the use of the Q-switch Nd: YAG laser with our suggested treatment protocol and irradiation parameters resulted in complete disinfection of the contaminated titanium discs. Therefore, the disinfection of titanium discs contaminated with a multi-species biofilm found in peri-implantitis may be promising for the management of peri-implantitis.

Identical letters indicate the absence of a statistically significant difference, while the difference in letters indicates a statistically significant difference. *p*-value < 0.0001.

The null hypothesis was rejected because there is a significant difference between the level of disinfection of contaminated and untreated titanium surfaces (Group C) and those that have been contaminated and laser-treated (Group L) having total surface disinfection.

## 4. Discussion

The Nd: YAG laser uses a clear, solid, crystalline medium that emits radiation in the infrared region of the electromagnetic spectrum, with a wavelength of 1064 nm. The Nd: YAG laser is a fiberoptic delivering contact lasers that generate a free-running pulsed beam of energy [24,25]. It is well established that the physical properties of each laser wavelength influence the level of absorption and interaction with titanium, tissues, and the bacteria [25]. Different wavelengths were being investigated in the literature concerning laser-assisted management of PI, including the 980 and 810 nm diode laser, the Er: YAG, Er, Cr: YSGG, and the Nd: YAG [26,27].

Regarding the comparison of our findings with the current evidence found in the literature, a previous study of Namour et al. [21] demonstrated that similar laser irradiation conditions to those used in our study using the Q-Switch Nd: YAG laser led to a total detachment of all organic and non-organic (calculus) elements, and therefore, a total elimination of calculus, organic, debris, germs, toxins, and any carbonic element. Another recent study by Namour et al. [22] showed that irradiation conditions similar to the conditions used in our study can lead to the total elimination of multi-species biofilm from irradiated titanium surfaces [22]. The results of our study are in line with these two previously published studies. Our study confirmed that the same irradiation conditions by Q-switched Nd: YAG laser have led to the complete disinfection of irradiated titanium surfaces with an average of TBC of 0.000 CFU/mL similar to sterile titanium discs (0.000 CFU/mL). Consequently, our study can be considered as a promising procedure, as the total disinfection of the cleaned titanium surface may lead to a new biocompatible surface, which is one of the most important conditions to achieve successful osseointegration after any PI treatment.

In addition, the literature shows that the bactericidal effect and the safety of irradiation on titanium discs varies significantly between wavelengths and between treatment protocols. In this context, Misischia et al. [28] demonstrated that the CO_2_ and Er, Cr: YSGG laser presented a disinfection value of 96%. In our study, the Q-switch Nd: YAG laser resulted in a total disinfection of titanium-irradiated surfaces.

Concerning the temperature increase in laser-irradiated titanium surfaces, Geminiani et al. [29] revealed that the 980 nm diode laser whether in continuous or pulsed mode can result in a critical increase in temperature that is above the threshold of alveolar bone damage and therefore cannot be indicated for the treatment of contaminated titanium surfaces [29]. In contrast, the study of Monzavi et al. [30] using the Er: YAG laser showed that this wavelength, according to their parameters, is safe. In fact, the heat generated with a different cooling system of the Er: YAG laser was always lower than 5 °C [30]. Hence, it appears that the Er: YAG laser can be considered to be safe for implant surface irradiations. Kushima et al. [31] showed that the 808 nm wavelength of diode laser application with irradiation of 20 s, 1 W, and 50 Hz in contact mode will result in increased zirconia and titanium temperature above 10 °C and therefore is harmful for the surrounding implant bone [31]. However, the irradiation conditions used in the study of Namour et al. [21] have shown that the Q-switch Nd: YAG laser, under irradiation conditions similar to those used in our study, can be considered safe for use on titanium surfaces. In fact, the increase in temperature was around 1 °C, which is known to be below the threshold of bone damage [21]. We used similar irradiation conditions in our study. Finally, it can concluded form the literature that the Er: YAG and the Q-switch Nd: YAG seem to be to most suitable wavelengths to be used in the management of PI [21,22,26,27,30].

We selected the Q-switch Nd: YAG laser to be investigated in our study for several reasons. First, it offers an irradiation with very short pulses down to the nanoseconds, which has been shown to lead to a minimal increase in temperature (<1 °C) of the irradiated titanium surfaces [21]. Second, the Q-switch wavelength has an interesting cleaning effect on titanium surfaces [22], an ability to completely destroy biofilm [22], and a high disinfection potential. Our study resulted in the total disinfection of the irradiated titanium surfaces. The third reason is the ability of the Q-switch Nd: YAG laser to produce a surface cleaning and disinfection effect in a short irradiation time (1–2 s) and in a single irradiation of each targeted area, unlike at other wavelengths. In fact, Nejem Wakim et al. [32] showed that the surface cleaning by the Er: YAG laser was not effective after one single irradiation, and that it is necessary to perform multiple irradiation passages on the same surface area of the titanium in order to have a significant surface cleaning [32]. In addition, the use of diode lasers (810 and 980) has shown excessive heating during irradiation even in pulsed mode [29,31], which can present a serious limitation of these wavelengths on titanium surfaces during PI treatments [29,31]. For these reasons, the diode and erbium lasers were not chosen for this study, and the Q-switch Nd: YAG laser was selected.

Several other treatment procedures are proposed in the literature. Photobiomodulation (PBM) and photodynamic therapy (PDT) seem to have promising bactericidal effects that can optimize the treatment of PI and periodontitis with no reported side effects [33,34]. In this matter, Al-Askar et al. [35] showed that the combination of PBM and PDT can reduce the peri-implant inflammatory parameter; however, no significant difference was reported in the pocket depth when compared to the conventional treatment of PI that is effective in the non-surgical treatment of PI [35]. Unlike our study, no bacteriological study was made to elucidate the disinfection potential of the suggested approaches. Apart from the use of the laser, additional approaches are being studied to optimize the results of the non-surgical and/or surgical management of PI such as probiotic Lactobacillus Reuteri [36,37]; chlorhexidine is considered as the molecule of choice [14,38].

Based on the results of the literature and the previous studies of Namour et al. [21,22], the irradiation conditions used in our study could be considered safe for periodontal tissues due to the very low heat generation around the targeted titanium surfaces. Moreover, the increase in temperature may also lead to possible damage of the topography of the titanium surfaces after laser irradiations. The irradiation conditions used in our study were theoretically evaluated in the study of Namour et al. [21]. In fact, the heat diffusion length is only dependent of the thermal diffusivity coefficient of titanium and of the $\sqrt{\tau }$ of irradiation time independent from the laser pulse energy [21]. The depth of the heat penetration in irradiated titanium surfaces was theoretically calculated to reduce the heat diffusion in the depth of titanium surfaces to less than 1 µm (according to the theorical modeling, the values are approximately 0.196 µm) [21] by the reduction of the pulse duration to a few nanoseconds. The surface melting is at that depth less than 1 µm, which is much smaller than the size of the implant surfaces porosity [21], and therefore, the shapes of the titanium surface microstructure will be preserved. The SEM examinations confirmed that this irradiation did not cause any topographical or structural damage on the titanium porosity surface [21]. Furthermore, it was demonstrated that the increase in temperature obtained at the titanium surface surrounding the targeted areas cannot provoke a cumulative effect of heat because the laser pulse separation in time (resting time) was two orders of magnitude larger than the pulse duration, allowing heat dissipation and reducing the heat cumulation effect [21].

The results of our study demonstrated that the use of the Q-Switch Nd: YAG laser under specific and precise irradiation conditions is capable of producing the total disinfection of contaminated titanium surfaces. This disinfection ability may be explained by the significant high temperature increase at irradiated surfaces leading to the complete elimination or destruction of bacteria by a desorption process. In addition, the desorption process produces a thermal degradation of contaminants that led to the removal of any adsorbed molecules. The absorbed optical laser energy is thermalized in the substrate of contaminants, leading to the desorption process and elimination of adsorbed substrates [21]. The very short pulses of the laser beam in nanoseconds can increase the temperature on the superficial surface of the titanium allowing the desorption and the removal of all contaminants [21]. This desorption process was able to eliminate all substrates including organic elements (toxins, debris, and biofilm) [21,22]. In our study, the superficial increase in temperature and the desorption process have led probably to the total disinfection of irradiated surfaces. Therefore, the use of the Q-switch Nd: YAG laser at specific irradiation conditions may be considered for any future pilot clinical study that focuses on the management of periimplantitis.

The results obtained in this study were very promising, since a total disinfection was perceived. For this reason, future in vivo and clinical studies using our irradiation conditions are needed to demonstrate the harmlessness and the clinical efficiency of our irradiation protocol in the treatment of peri-implantitis.

## 5. Conclusions

In conclusion, the present study confirmed that the Q-Switch Nd: YAG laser under our specific irradiation conditions can provide a total disinfection of the contaminated titanium surfaces.

## Figures and Tables

**Table 1:** Mean with standard deviation of the TBC of microbiological assessments in the three groups. The TBC is expressed in CFU/mL.

	Group L	Group C	Group S
sample size	24	24	24
mean value	0.000 ^A^	4767 ^B^	0.000 ^A^
std deviation	0.000	1924	0.000
std. error	0.000	555.3	0.000

## Data Availability

Not applicable.
